# HIV sexual risk behavior and preferred HIV prevention service outlet by men who have sex with men in Nigeria

**DOI:** 10.1186/s12913-019-4108-z

**Published:** 2019-04-27

**Authors:** Godwin Emmanuel, Morenike Oluwatoyin Folayan, Bartholomew Ochonye, Paul Umoh, Bashiru Wasiu, Mercy Nkom, Apera Iorwa, James Anenih

**Affiliations:** 1 Heartland Alliance International, Abuja, Nigeria; 2 New HIV Vaccine and Microbicide Advocacy Society, Lagos, Nigeria; 3 Department of Child Dental Health, Obafemi Awolowo University, Ile-Ife, Nigeria; 4 West African Infectious Diseases Institute, Abuja, Nigeria; 5 National Agency for the Control of AIDS, Abuja, Nigeria

## Abstract

**Background:**

The study objectives were to identify differences in HIV sexual risk behavior of men who had sex with other men (MSM) resident in urban and rural Nigeria, their perspectives on need for HIV prevention services and perceived barriers and facilitators to access of HIV prevention services in private, public and peer-led health facilities.

**Method:**

Data were collected from MSM resident in urban and rural parts of River and Kaduna States. Qualitative assessment sought perspectives on barriers and facilitators of MSM uptake of HIV prevention services. In addition, a questionnaire was administered to seek information on HIV sexual risk behaviors (sexual abuse, age of sexual debut, multiple sexual partners and use of condom at last sexual intercourse), willingness to use and perceived barriers to access of HIV prevention services in public, private and peer-led health facilities, and willingness to use and perception about availability of structural intervention services. Differences in HIV sexual risk behaviors by residential location, and associations between sexual risk behavior and willingness to access HIV prevention services were determined.

**Results:**

More MSM resident in urban than rural areas engaged in three or more HIV sexual risk behaviors (25.9% vs 8.7%; *p* = 0.02). More respondents were willing to access HIV prevention service provided through peer-led health facilities. Less than 35% of respondents identified non-availability of free services as a barrier to HIV prevention service access in the three types of health facilities. More MSM with multiple sexual risk behaviors were willing to access services promoting mental and psychosocial health (*p* < 0.001), HIV positive peer support programs (*p* = 0.002) and training on human rights and paralegal services (p < 0.001). Respondents opined that services that assured confidential HIV testing and mitigated structural drivers of HIV infection for MSM provided through peer-led facilities, will increase MSM’s uptake of HIV prevention services.

**Conclusion:**

HIV risk reduction intervention services differentiated by rural and urban residence, may be needed for MSM. Services provided through peer-led facilities, that include mental and psychosocial health care, peer support, human rights and paralegal services will likely increase its use by MSM with more HIV sexual risk behaviors.

## Introduction

Nigeria has the second highest burden of HIV in the world after South Africa, representing 9.0% of the global burden of infection [1]. Key populations, namely men who have sex with other men (MSM), female sex workers and people who inject drugs, contribute significantly to the prevalence of HIV infection in the country [2]: HIV transmission in Nigeria is driven by networks involving key populations. Populations at high-risk for HIV infection constitute 3.4% of the Nigerian population (it is estimated that there are 11,748 MSM in Nigeria [3]), yet 40.0% of new HIV infections in the country is attributable to key populations and their sex partners [3].The national HIV prevalence for MSM is 22.9% [4].

The national HIV prevention response program introduced the Minimum Prevention Package of Intervention (MPPI) [5] in an effort to improve access of the general population and key populations to comprehensive HIV prevention services in Nigeria [6–8]. The MPPI promotes access of MSM to a minimum of three behavior change communication contacts. Exposure to a minimum of three sessions help reinforce messages, and improve access to condoms and lubricants, quarterly testing for HIV infection, STI management and structural programs that reduce stigma and promotes behavior change maintenance. MPPI improved the quality of HIV prevention service delivery [9].

A study conducted by Heartland Alliance named the Enhancing Key Population Intervention in Nigeria through Capacity Development (EKPIN) project [10], identified that a significant percentage of MSM (6.6%) had a history of forced sexual initiation and used and abused psychoactive drugs (23.6%). Forced sexual initiation increases the risk for HIV infection due to associated mental distress that results in deregulation of the protective immune system [11, 12], engagement in high risk behaviors such as multiple sex partners and exchange of sex for money, and or use of psychoactive drugs due to heightened sexual behavior and easy arousal, and low self-esteem [13, 14]. Also, persons who has a history of forced sex initiation have low competency to refuse unwanted sex, and negotiate safe sex [15]. Associated post-traumatic stress disorders increased the use psychoactive substances, further reducing the ability to negotiate safe HIV preventive behaviors [16, 17]. Some MSM in Nigeria may therefore need services that will enhance their mental health. The current MPPI package does not include structural interventions to address the mental health needs of MSM.

Also, the unfavorable legal environment for MSM through the introduction of the Same Sex Prohibition Act [18] mitigates access of MSM to HIV prevention services: the law drove MSM into hiding with deleterious impact on access to HIV prevention and treatment services in a peer-led facility [19]. Although Staff in selected public and private hospitals across Nigeria are trained to provide key population friendly services [20], concerns about stigma, discrimination, privacy and confidentiality had limited use of these facilities by MSM [10]. Drop-in-centers were established through the USAID funded ‘Strengthening HIV Prevention Services for Most-at-Risk Populations’ project [21] to facilitate access of MSM to HIV testing and STI syndromic management. There is however no formal evaluation of MSM’s perspective on challenges with HIV prevention service access in the public, private and peer-led facilities; and how to improve access of MSM to HIV prevention services.

This study tried to address this gap. Specifically, it identified the HIV sexual risk behaviors of MSM resident in urban and rural Nigeria and determined if there were differences in the prevalence of these behaviors per residential location. Also, it assessed the perception of MSM about their need for HIV prevention services; perceived obstacles and facilitators to uptake of HIV prevention services in private, public and peer-led health facilities; and the association between HIV sexual risk behaviors and the need for structural intervention services. We concluded by describing a service delivery model that would help improve uptake of HIV prevention programs by MSM in Nigeria.

## Methods

### Study design

The study used a qualitative and quantitative method to generate data. The study used both methods to generate data for triangulation of findings. The qualitative data helped to corroborate the findings from the quantitative data. It also provided context relevant explanation to some of the findings from the quantitative data. Qualitative data were collected using in-depth interviews (IDI) and key informant interviews (KII) conducted with key opinion leaders in the MSM community, and focus group discussions (FGD) with MSM. Quantitative data were collected through interviewer administered structured questionnaire.

### Study population

Study participants were MSM resident in urban and rural Nigeria. Study participants had to be 18-years-old or older and self-identified as a MSM. The identified seeds had served as peer educators in structured HIV prevention services programs for MSM. MSM involved in the development of the study protocol were excluded from study participation.

Study participants were recruited from two states in Nigeria - Rivers and Kaduna States. These states had ongoing donor (PEPFAR, Global Fund and World Bank) funded programs that supported the access of key populations to HIV prevention services. The two states were selected to enhance the geographical diversity of study participants thereby increasing the representativeness of the data. Rivers State is located in Southern Nigeria while Kaduna State is located in Northern Nigeria.

### Recruitment for quantitative study

A convenient sample of 300 MSM (150 per state was proposed for the quantitative study: 100 (50 per state) from rural area and 200 (100 per state) from urban areas. In River State, study participants were recruited from the riverine Bonny Island (rural area) and Port Harcourt (urban area). In Kaduna State, study participants were recruited from Samineka and Kanfanchan (rural areas), and Kaduna (urban area).

The snowball approach was used for recruitment. Initial study contacts were identified from civil society organizations working with MSM in the target study sites. Five initial contacts in the rural areas and 10 initial contacts in the urban areas were identified as seeds. These contacts were peer educators enlisted on the Heartland Alliance peer education programs implemented in the target states. The seeds invited two MSM each in the first wave of recruitment. The MSM identified in the first wave of recruitment invited two peers each for the second wave of recruitment. Efforts were made to ensure the selected seeds were identified from various hotspots highlighted in the epidemic appraisal in the target states and from different age groups [22]. This helped ensure geographical diversity of participants, and prevented recruitment of respondents from a single cluster. The use of snow balling technique is appropriate for the recruitment of hard to reach populations [23], and the use of appropriate strategies enhances the diversity of study participants [24].

### Recruitment for qualitative study

Ten FGD were conducted (five per state: two in the rural area, two in the urban area, and one with MSM who had not publicly disclosed their sexual orientation resident in Rivers State). Each FGD included 10 participants. Six KII (three from each state) were conducted with MSM who had accessed the MPPI through an MSM intervention program implemented over the last three years preceding the conduct of the study. Thirty IDI (15 in each state) were conducted with MSM who were not willing to participate in FGD but were identified as key opinion leaders in the community. In total, 136 MSM were recruited for the qualitative study. These participants were not included in the quantitative study.

Recruitments for the FGD and IDI were made through contacts with non-governmental organizations working with MSM in the target States. Participants were randomly (every 5th person) selected from the list of clients at the organization, and invited by the organization to participate in the FGD. Those that declined to participate in FGD were invited for IDI. Recruitment of study participants continued until the number of participants required for the FGD and IDI were reached. Invitees for the KII were purposefully selected. They were MSM who had had accessed the MPPI through an MSM intervention program implemented over the last three years preceding the study. They were also identified to be key opinion leaders in the community.

### Study procedure

Field workers were selected based on their competence and experience working with MSM, and trained on the study protocol and use of the data collection tools. The IDI solicited the participants’ perspectives about structural issues that affect the access of MSM to HIV prevention services. This included discussions on challenges with operationalization of the MPPI for MSM, suggestions for improving its operationalization and how to optimize uptake of the MPPI by MSM. For the KII, each participant was interviewed about their experiences and perspectives on enablers and barriers for HIV prevention service access and uptake, and how the services could be improved. FGD were conducted to seek the views of discussants on the importance of provision of HIV prevention services for MSM and how to improve access of MSM to HIV prevention services. All interviews and FGDs were conducted in a private room in the offices of the organizations that provided contacts. Participants who came for the FGD were reimbursed N2000 (approximately $11.50) for transport, and were provided refreshment. All participants were provided with written information about the project.

For the IDI and KII, the field worker greeted the participant, explained the purpose of the interview and obtained consent before proceeding with the data collection and audio-recording the sessions. At the end of the IDI and KII, participants were thanked for their time, and reimbursed for transportation. Immediately following each IDI and KII, the interviewers wrote a detailed debriefing note and filled out a one page debriefing form that listed some basic statistics about the session and a summary report of the interview. The FGD also took the same format except for the presence of a note-taker who wrote the debriefing notes. Participants were encouraged to keep all information shared at the session confidential.

### Study questionnaire

The study questionnaire was divided into eight sections. The first section generated information on participants’ profile (age and level of education, history of use of psychoactive substances and history of sexual intercourse). The second section generated information on the HIV sexual risk behavior – history of sexual abuse, age of sexual debut, multiple sexual partners and use of condom at last sexual intercourse. The third section generated information on willingness to use HIV prevention services. The fourth, fifth and sixth sections generated information on the perceived barriers to access of HIV prevention services by MSM when using public, private and peer-led facilities respectively. The seventh section enquired about willingness of MSM to use services that provided structural interventions. The eight section elicited information on perception of the availability of structural intervention services. The tool used for the study had been used in a prior study conducted to generate similar information from MSM, female sex workers, and people who inject drugs resident in four States in Nigeria [10].

Sixteen questions were asked on the willingness of respondents to use HIV prevention services. Responses options to each question were ‘very willing’, ‘neutral’ or ‘not willing’. Table 2 highlights each of the 16 questions asked.

Ten questions were asked on perceived barriers to access of HIV prevention services when using public, private and peer-led facilities respectively. Respondents were asked to respond to a ‘yes’ if they perceived listed factors would serve as a barrier to access of HIV prevention services in public, private or peer-led facilities, or a ‘no’ if the factors would not serve as a barrier to accessing HIV prevention services in those institutions. These questions are highlighted in Table 3.

Questions were asked on willingness to use services that provided eight structural interventions and respondents’ perceived availability of these eight services. Response option for willingness to use services ranged from ‘very willing’, ‘willing’, ‘seldom willing’ and ‘not willing’. Response option on perceived availability of services ranged from ‘always available’, ‘available’, ‘seldom available’ and ‘not available’. For analysis purposes, the responses ‘very willing’ and ‘willing’ were collapsed into a single response (willing) and the responses ‘seldom willing’ and ‘not willing’ were collapsed into a single response (not willing). Similarly, the responses ‘always available’ and ‘available’ were collapsed into a single response (available) and the responses ‘seldom available’ and ‘not available’ were collapsed into a single response (not available). These questions are highlighted in Table 4.

The study instruments were developed in English. All the interviews and FGDs were conducted in English. The questionnaires were also administered in English. While it might be useful to translate entire questionnaires into local languages, this would be impractical given the multiplicity of languages in Nigeria. Instead, key words and phrases, especially sensitive ones, were translated in the languages of each selected community and collated as a list generated during the training of interviewers. Interviewers used this material as a reference when in the field. A similar technique was successfully used past studies on sexual and reproductive health in Nigeria [25–27]. Participants for the FGD were encouraged to share their opinions in languages they felt comfortable with.

### Data analysis for quantitative data

The proportion of respondents who engaged in HIV sexual risk behaviors, those willing to use HIV prevention services and those who identified specific barriers to access of HIV prevention services in public, private and peer-led facilities were analyzed. Also, the proportion of those who were willingness to use different structural intervention services and those who perceived those structural intervention services were available were analyzed. The association between HIV sexual risk behavior and willingness to access structural interventions was also determined. Pearson’s chi-square and Fischer’s Exact test were used to test significance of associations where appropriate. Statistical significance was established at *p* ≤ 0.05.

### Data analysis for qualitative data

Transcripts from audio recorded and summary notes from the IDI, KII and FGDs were analyzed using a grounded approach. Inductive thematic analysis was conducted to identify salient themes. Themes that emerged from the interviews and discussions on perception of need for HIV prevention services, challenges MSM encounter in accessing HIV prevention service, ways to address the challenges identified, and proposals for improving the current service delivery models for the MPPI were highlighted. The findings of the survey and the qualitative data were triangulated.

### Ethical considerations

The study protocol received ethics approval from the Jos University Teaching Hospital Health Research Ethics Committee (JUTH/DCS/ADM/127/XIX/6261). Written consent was also obtained from all study participants.

## Results

### Socio-demographic profile of study participants

Two hundred and ninety nine participants were recruited for the study constituting 99.7% of the proposed sample size. This included 295 (98.6%) males and 2 (0.7%) transgender persons. Table 1 highlights the socio-demographic profile of study participants by residential location. There was no significant difference in the gender, history of sexual intercourse, and injecting drug use of MSM resident in urban and rural areas. More MSM resident in urban areas had tertiary education (*P* < 0.001) and used psychoactive drugs (*p* = 0.003) than MSM resident in rural areas.

**Table 1:** Profile of respondents by area of resident (*N* = 299)

S. no	Variables	MSM Rural (*n* = 95)	MSM Urban (*n* = 204)	X^2^	*p* value	Total
1	Sex					
	Male	95 (100%)	202(99.0%)	0.94	1.00	297(98.6%)
	Transgender	0 (0.0%)	2 (1.0%)			2 (0.7%)
2	Educational Level					
	None	5 (5.3%)	4 (2.0%)	34.77	< 0.001	9 (3.0%)
	Primary	51(53.7%)	47 (23.0%)			98 (32.8%)
	Secondary	37(39.0%)	138(67.6%)			175(58.5%)
	Tertiary	0 (0.0%)	13 (6.4%)			13 (4.3%)
	No Response (8–1.3%)	0 (0.0%)	2 (1.0%)			4 (1.4%)
3	Age range					
	18-24 yrs	27(28.4%)	73 (34.8%)	2.70	0.44	100(33.4%)
	25-34 yrs	43(45.3%)	74 (36.3%)			117(39.1%)
	35-45 yrs	18(18.9%)	44 (21.5%)			62 (20.7%)
	46+	7 (7.4%)	13 (6.4%)			20 (6.7%)
4	Ever used drugs					
	Yes	10(10.5%)	47 (23.0%)	9.13	0.003	57 (19.1%)
	No	80(84.2%)	125(61.3%)			205(68.6%)
	No response	5(5.3%)	32 (15.7%)			37 (12.3%)
5	Ever injected drugs					
	Yes	2 (20.0%)	8 (17.0%)	0.05	1.00	10 (17.5%)
	No	8 (80.0%)	39 (83.0%)			47 (82.5%)
6	History of sexual intercourse					
	Yes	91(95.8%)	201(98.5%)	1.35	0.56	292(97.7%)
	No	0 (0.0%)	3 (1.5%)			3 (1.0%)
	No response	4 (4.2%)	0 (0.0%)			4 (1.3%)

### Sexual behaviour

The mean age at first sexual debut was 17.1 years + (4.7) (95% CI: 17.3–18.5). The age of sexual debut ranged from 5 years to 35 years. The modal age was 20 years. The mean age of sexual debut for MSM resident in urban areas was 18.9 + (4.5) (95% CI: 18.2–19.6) and that for MSM resident in rural areas was 15.9 + (4.3) (95% CI: 15.0–16.8).

**Fig. 1:**
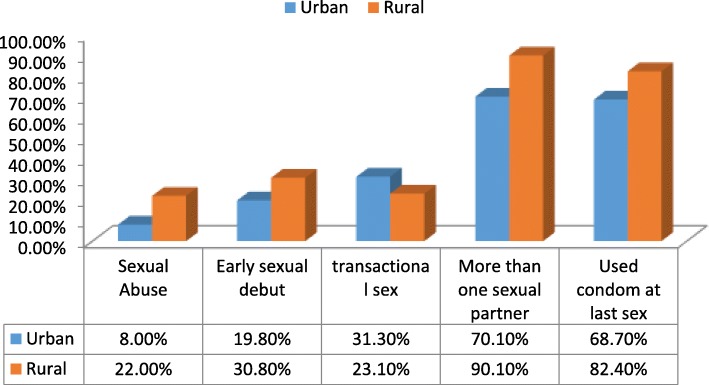
HIV Sexual risk profile of MSM by residential area

Figure 1 highlights the sexual behavior of study participants. Nineteen (6.5%) MSM had no HIV sexual risk behavior. More MSM in urban than rural areas engaged in three HIV sexual risk behavior (25.9% vs 8.7%; x^2^ = 9.43; *p* = 0.02), transactional sex (x^2^ = 1.61; *p* = 0.20) and did not use use condom at last sexual intercourse (x^2^ = 5.58; p = 0.02). More MSM in the rural than urban areas experienced forced sexual initiation (x^2^ = 12.16; *p* < 0.001), had early sexual debut (x^2^ = 4.35; *p* = 0.04), and have more than one sexual partner (x^2^ = 15.09; p < 0.001).

### Willingness to access HIV prevention services

Table 2 highlights the proportion of respondents willing to access HIV prevention services. More than 89% of the respondents were willing to receive regular behavior change communications, condoms, lubricants and visit clinics for STI check-ups. Only 50.3, 61.1 and 79.7% of respondents were willing to access these services in a public, private and peer-led health facilities respectively.

**Table 2:** Proportion of MSM in Nigeria Showing Willingness to receive HIV Prevention Services (N = 297)

How willing are you to:		Very Willing	Neutral	Not Willing
Q101	Attend regular meetings organized to discuss HIV related issues?	277 (93.2%)	15 (5.1%)	5 (1.7%)
Q102	Allow people to ask you questions about your risk behavior?	266 (89.6%)	22 (7.4%)	9 (3.0%)
Q103	Follow a plan to help you address your HIV risk?	292 (98.3%)	1 (0.3%)	4 (1.4%)
Q104	Receive and use condom?	287 (96.6%)	6 (2.0%)	4 (1.4%)
Q105	Receive and use lubricant?	285 (95.9%)	7 (2.4%)	5 (1.7%)
Q106	Visit clinics for STI check-ups?	272 (91.6%)	13 (4.4%)	12 (4.0%)
Q107	Go to public clinic to get HIV related services?	154 (51.9%)	56 (18.9%)	87 (29.2%)
Q108	Go to private clinic to get HIV related services?	181 (61.0%)	39 (13.1%)	77 (25.9%)
Q109	Go to peer led clinic to get HIV related services?	237 (79.8%)	48 (16.2%)	12 (4.0%)
Q110	Attend public clinics if accompanied by peers?	152 (51.2%)	42 (14.1%)	103 (34.7%)
Q111	Attend private clinics if accompanied by peers?	163 (54.9%)	40 (13.5%)	94 (31.6%)
Q112	Attend peer led clinics if accompanied by peers?	223 (75.1%)	29 (9.8%)	45 (15.1%)
Q113	Have peers facilitate access to services in public hospitals if you encounter difficulties?	185 (62.3%)	62 (20.9%)	50 (16.8%)
Q114	Have peers facilitate access to services in private hospitals if you encounter difficulties?	182 (61.3%)	43 (14.5%)	72 (24.2%)
Q115	Have peers assist in getting voluntary HCT?	246 (82.8%)	25 (8.4%)	26 (8.8%)
Q116	Have peers serve as drug adherence supporter if HIV positive?	231 (77.8%)	30 (10.1%)	36 (12.1%)

### Perceived obstacles to uptake of HIV prevention services in public, private and peer-led health facilities

Table 3 highlights barriers to access of HIV services by MSM in public, private and peer-led health facilities. More than 50% of the respondents identified eight of the 10 variables as obstacles to receiving HIV prevention services in public, private and peer-led health facilities. Less than 50% of the respondents identified distance as a barrier to accessing HIV prevention services at peer-led health facilities. Less than 35% of respondents identified non-availability of free services was as a barrier to access of services in public, private and peer-led health facilities.

**Table 3:** ^a^Analysis of the Differences in the Proportion of MSM Responding Positively to Factors that May Make Services Provided by Public, Private and Peer-Led Health Facilities Not Accessible to MSM (*N* = 297)

Factors		Public	Private	Peer
A	Non-availability of free services	70 (23.6%)	91 (34.1%)	71 (23.9%)
B	The distance of service delivery points to home	169 (56.9%)	180 (60.6%)	140 (47.1%)
C	Lack of knowledge about HIV by provider	199 (67.0%)	157 (52.9%)	160 (53.9%)
D	Lack of friendly facilities	226 (76.1%)	222 (74.7%)	186 (62.6%)
E	Inadequate Information specific to MSM	221 (74.4%)	211 (71.0%)	167 (56.2%)
F	Inability to provide HIV counselling services	200 (67.3%)	197 (66.3%)	175 (58.9%)
G	Stigmatization by providers	243 (81.8%)	220 (74.1%)	205 (69.0%)
H	Availability of service provider to manage stigma related crisis	173 (58.2%)	175 (58.9%)	157 (52.9%)
I	Inability to address police harassment	221 (74.4%)	213 (71.7%)	189 (63.6%)
J	Inability to provide comprehensive services in same place (one stop shop)	173 (58.2%)	213 (71.7%)	175 (58.9%)

^a^Respondents were required to identify if each of the factors was a barrier for accessing services in the three types of service outlets. The table highlights the proportion of those who identified each of the factors as barriers

### Access to structural intervention that sustains HIV prevention behaviors

Table 4 highlights responses on the willingness to access programs that provide structural supports to enhance behavioral changes. More than 79% of respondents were willing to access the following structural interventions – income generating activities, legal services, services for mental health and psychosocial support, positive peer support programs, and adherence support for use of antiretroviral therapy. However, fewer MSM who were willing to accesses these services identified that these services were available.

**Table 4:** Analysis of the Differences in Number of MSM Willing to use HIV Prevention Services and Perception on Availability of the Services in Nigeria (N = 297)

Factors		Willing	Available
A	How willing are you to participate in economic empowerment/income generating activities to address transactional sex?	251 (84.8%)	77 (26.1%)
B	How willing are you to receive legal services to address discrimination based on your sexual orientation?	258 (86.8%)	62 (21.0%)
C	How willing are you to receive social justice for discrimination based on your sexual orientation?	252 (84.8%)	98 (33.0%)
D	How wiling are you to receive training on fundamental human rights and paralegal services?	280 (94.2%)	82 (27.6%)
E	How willing are you to access other health services health promoting services e.g. mental health, psychosocial services?	260 (87.6%)	136 (46.3%)
F	How willing are you to participate in a HIV Positive Peer Support program?	235 (79.1%)	180 (60.8%)
G	How willing are you to receive support services for adherence to ART?	248 (83.8%)	194 (65.3%)
H	How willing are you to be accompanied in referral for ART services?	238 (80.7%)	177 (60.2%)

### Association between sexual risk behavior and willingness to access structural interventions

Table 5 shows the association between sexual risk behavior and willingness to access structural intervention programs. Significantly more MSM with HIV sexual risk behaviors were willing to access services health promoting mental and psychosocial health (*p* < 0.001), receive training on human rights (*p* < 0.001) and participate in HIV positive support group programs (*p* = 0.002).

**Table 5:** Association between HIV Sexual Risk Behavior and Willingness to Access Structural Interventions by MSM in Nigeria (*N* = 297)

S.no	Variables	Respondents with at least one HIV sexual risk behavior (*N* = 297)		X^2^	*P* value
		Yes (278)	No (19)		
Q401a	How willing are you to participate in economic empowerment/income generating activities to address transactional sex?	237 (85.3%)	14 (73.7%)	1.82	0.18
Q401b	How willing are you to receive legal services to address discrimination based on your sexual orientation?	241 (86.7%)	17 (89.5%)	0.12	1.00
Q401c	How willing are you to receive social justice for discrimination based on your sexual orientation?	235 (84.5%)	17 (89.5%)	0.34	0.75
Q401d	How wiling are you to receive training on fundamental human rights and paralegal services?	273 (98.2%)	7 (36.8%)	124.08	< 0.001
Q401e	How willing are you to access other health services health promoting services e.g. mental health, psychosocial services?	252 (90.6%)	8 (42.1%)	34.43	< 0.001
Q401f	How willing are you to participate in a HIV positive peer support program?	224 (80.6%)	11(57.9%)	5.54	0.02
Q401g	How willing are you to receive support services for adherence to ART?	234 (84.2%)	14 (73.7%)	1.42	0.23
Q401h	How willing are you to be accompanied in referral for ART services?	224 (80.6%)	14 (73.7%)	0.53	0.47

### Factors that could facilitate or serve as barriers to HIV prevention service access by MSM at the health facilities

Participants who took part in the FGD, IDI and KII identified that uptake of HIV prevention services will increase when service recipients are assured of confidential HIV testing. Also, a participant identified HIV self-testing was a strategic approach to assuring confidential HIV testing:

On your own, you can conduct your test if you are well trained. This is better and more confidential than going out. **…**FGD KD 003

Participants also opined that the large number of patients at the public hospitals coupled with hasty review of patients by physicians, discourages MSM from using public health facilities and explains their preference for private health facilities. However, the cost of accessing care at private hospitals was a deterrent for use of private hospitals. One of the interviews noted:

So many of my friends that I referred to the community centre who told they will have free medical services always come back complaining that they were referred to another hospital for routine test and were heavily taxed. If the community centre can have comprehensive services it will help us more. ……. KII 06

### Approaches to improve MSM uptake of MPPI

Discussants acknowledged that the MPPI model promoted access to behavioral and biomedical interventions. It was however unable to effectively mitigate structural drivers of HIV infection for MSM. Critical HIV prevention needs for MSM were interventions that promote skills acquisition to enable community members generate income and become self-reliant. More MSM needed information about MPPI service delivery points and how to access them. Peer-led facilities are best able to provide HIV prevention services for MSM: these facilities are more hospitable and handle information more confidentially. These attributes foster continuous use of the services. Below is a quote by one of the interviewees.

The hospitality I receive each time I visit the community centre is commendable, the Peer Education system is one of the programs I enjoyed most and Issue of confidentiality is nothing for me because the peer educators have earn my trust… KII 04

## Discussion

There were three key findings from the study. First, there are differences in the HIV sexual risk behaviors of MSM resident in urban and rural areas: more MSM in urban than rural areas had three or more HIV sexual risk behaviors and were less likely to use condom at last sexual intercourse; while more MSM in the rural than urban areas had a history of forced sexual initiation, had earlier age of sexual debut, and had more than one sexual partner. Second, more MSM were willing to access HIV prevention service provided through peer-led health facilities. Factors that deter MSM from using public health centers were service providers’ poor knowledge about HIV and MSM health issues, non-friendly services, stigmatization by service providers and inability of public health facilities to prevent police harassment. Third, significantly more MSM with multiple sexual risk behaviors were willing to access mental and psychosocial health services, HIV positive peer support programs and training on human rights and paralegal services. Most MSM willing to access these services felt they were not available.

One of the strength of this study was the recruitment strategies. The geographical and age diversity of the seeds helped with the recruitment of a wide range of MSM for this study. The findings are therefore applicable to MSM in Nigeria in general; and provide evidences to generate new hypothesis to further study challenges MSM face in accessing HIV prevention services in Nigeria.

The study however has a limitation. A sample size was not determined for the study thereby limiting the ability to determine if the study was powered to address the study objectives. Also the response rate the query on drug use by MSM resident in urban areas was 15.7% undermining the validity of the findings for this enquiry. We feel this may be due to sensitivity around police harassment and arrest of drug users, which we did not anticipate during the data collection process and therefore, possibly did not provide enough assurance for respondents on the confidentiality of their response on this enquiry in the way the question was framed. Despite these limitations, the study generated findings that are novel for Nigeria and are important for making strategic decisions about how to improve on the current HIV prevention interventions for MSM. There are four critical findings that can inform the (re)design of HIV prevention support services for MSM in Nigeria.

First, while more MSM were willing to access HIV prevention services offered through peer-led facilities, the number of these peer-led facilities in Nigeria are limited. Access to HIV prevention services for MSM need to be augmented by public and private hospitals. Addressing the barriers to accessing services in public and private clinics highlighted in this study can enhance not only the needs of MSM, but also possibly enhance update of services by members of the general public who also have similar complaints about the poor quality of services offered in public and private facilities in Nigeria [28]. Increasing HIV prevention service outlet options for MSM also helps increase support for the diverse populations of MSM to access services [29, 30].

Second, HIV prevention programs for MSM may need to be tailored to needs based on residential locations. MSM resident in urban areas will need interventions that support consistent use of condoms. In the rural area, efforts may need to focus on delaying the age of sexual debut; and reducing the risk for forced sexual initiation. Adolescents who experience forced sexual initiation are more likely to engage in high HIV sexual risk behaviors [17]. Also, unmanaged traumatic stress increases mental health disorders [31]. The silence about sexual violence faced by males in Nigeria is worse that the silence about rape that females face [32]. The silence about forced sexual initiation females face is being broken as a result of the movement for open discussion and punishment of rapists. Similar actions can help break the silence around rape of male children and adolescents.

Third, it is apparent that while the MPPI had been successful in promoting access of MSM to biological and behavioral interventions, it has not been successful in providing the structural interventions essential for behavior change maintenance: empowering MSM with skills to generate income reduces the likelihood of engaging in transactional sex, and multiple sexual partnering. Also, access to legal services enhances individual’s ability to address discrimination based on sexual orientation. These interventions are best provided by non-governmental organizations. Unfortunately, there are very few peer-led MSM organizations in the country that can provide structural interventions for MSM [33, 34]. The country needs more peer-led health facilities that provide these needed interventions for MSM.

Fourth, mental health support are needed for MSM in Nigeria [35]; and a large number of respondents were interested in accessing mental and psychosocial health services. Sexual minority stress increased the vulnerability of MSM to mental health problems [36], which in itself, constitutes a risk for HIV infection. The unsupportive legal, social, cultural and religious environment in Nigeria [35] increases MSM’s risk for mental health problems, further highlighting the urgent need for mental health service support for MSM. Peer support helps improve mental health [36]: it is a stress buffer, has a salutary effect on mental health and psychological well-being, and provides a form of social capital for network members [37]. Unfortunately, this service is not readily accessible by MSM in Nigeria. Civil society organizations need to be supported to implement peer support programs for MSM in Nigeria.

## Conclusion

A significantly large number of MSM are willing to access HIV prevention services through peer led health facilities. The current national MPPI program for MSM needs to be augmented with interventions that promote skills development for income generation, access to mental and psychosocial health services and peer support services. Differentiated HIV prevention interventions programs is needed for MSM resident in urban and rural areas. The capacity of civil society organizations can be built to address the structural interventions gaps for MSM identified in this study.

## Abbreviations

EKPIN: Enhancing Key Population Intervention in Nigeria through Capacity Development
FGD: Focus Group Discussion
HIV: Human Immunodeficiency Virus
IDI: In-depth Interview
KII: Key informant Interview
MPPI: Minimum Prevention Package of Intervention
MSM: Men who have Sex with Men
PEPFAR: Presidential Emergency Program for AIDS Relief
USAID: United States Agency for International Development
